# Not just sugar: Metabolic control of neutrophil development and effector functions

**DOI:** 10.1093/jleuko/qiae057

**Published:** 2024-09-02

**Authors:** Paul Ettel, Thomas Weichhart

**Affiliations:** 1 Institute for Medical Genetics, Center for Pathobiochemistry and Genetics, Medical University of Vienna, Währinger Straße 10, 1090 Vienna, Austria

**Keywords:** neutrophil, metabolism, immunometabolism, innate immunity

## Abstract

The mammalian immune system is constantly surveying our tissues to clear pathogens and maintain tissue homeostasis. In order to fulfill these tasks, immune cells take up nutrients to supply energy for survival and for directly regulating effector functions via their cellular metabolism; a process now known as immunometabolism. Neutrophilic granulocytes, the most abundant leukocytes in the human body, have a short half-life and are permanently needed in the defense against pathogens. According to a long-standing view, neutrophils were thought to primarily fuel their metabolic demands via glycolysis. Yet, this view has been challenged as other metabolic pathways recently emerged to contribute to neutrophil homeostasis and effector functions. In particular during neutrophilic development, the pentose phosphate pathway, glycogen synthesis, oxidative phosphorylation, and fatty acid oxidation crucially promote neutrophil maturation. At steady state, both glucose and lipid metabolism sustain neutrophil survival and maintain the intracellular redox balance. This review aims to comprehensively discuss how neutrophilic metabolism adapts during development, which metabolic pathways fuel their functionality and how these processes are reconfigured in case of various diseases. We provide several examples of hereditary diseases, where mutations in metabolic enzymes validate their critical role for neutrophil function.

**Figure:**
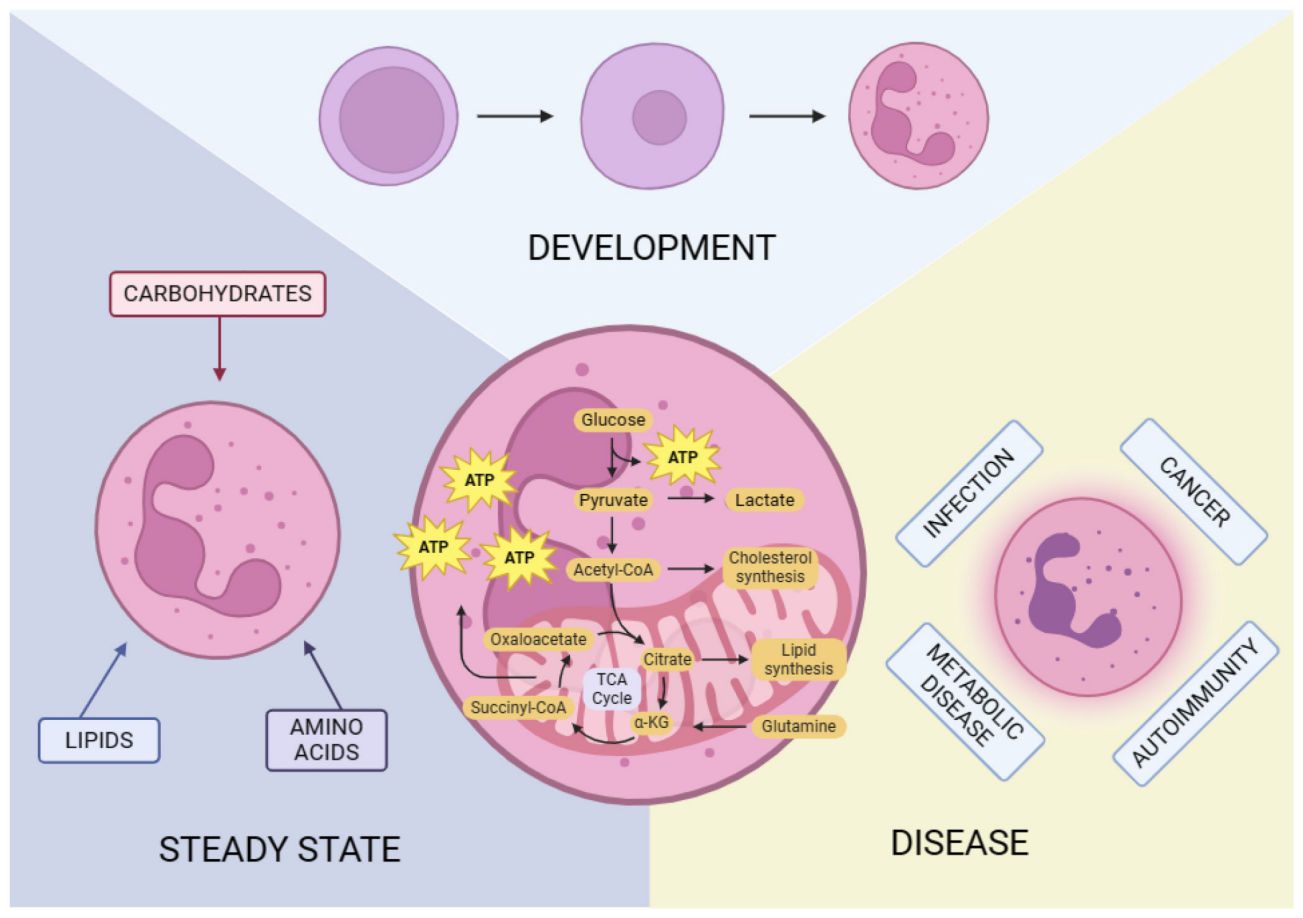

## Introduction

In recent years, it has become more evident that cellular metabolism in immune cells does not only provide building blocks for proliferation but can directly influence effector functions. This novel concept of “Immunometabolism” has changed the understanding of how the immune system must adapt metabolic needs to both support tissue homeostasis as well as in pathological challenges^1^. When specifically looking at the innate immune system, the poster child cell for the immunometabolism field have been mainly macrophages^2^, which precisely adapt their metabolism depending on the tissue microenvironment and in case of a disease^3,4^. More recently, however, a component of the innate immune system has caught more attention: the neutrophil. Neutrophils, that account for 50-70% of all leukocytes in peripheral blood and about 200 g in total mass in humans^5^ and 10-25% of blood leukocytes in mice^6^, were not in the focus of attention due to their short lifespan and proposed dependency on glucose and oxygen^7^. Yet an increasingly convincing argument can be made that neutrophils can also adapt their metabolism in specific settings^8,9^.

Neutrophilic granulocytes are most frequently coined as the first line of defense against invading pathogens^10^. The necessity of these cells to fight pathogens is most strikingly n in diseases like chronic granulomatous disease (CGD) or leukocyte adhesion deficiency (LAD). These hereditary diseases result in impaired neutrophil effector functions and frequently manifest in repeated infections and can be fatal at a young age if not treated at an early stage^11^. Neutrophils develop in the bone marrow and arise from granulocyte-monocyte progenitors (GMP). The developmental process in the bone marrow comprises of a proliferative and non-proliferative phase and takes 3 days in humans and 6 -7 days in mice to complete^12^. Traditionally, neutrophils are thought to have a life span of 24h, however, reports claim that the lifespan can be prolonged significantly^13^. In murine studies, a circadian rhythm was described for neutrophil dynamics in both blood and tissues, underpinning the proposed 24h time frame for neutrophil activity^14,15^. Functionally, neutrophils are equipped with three distinct effector functions: degranulation, phagocytosis, and formation of neutrophil extracellular traps (NETs) in a process termed NETosis^7,16^. NETs are extruded chromatin strands with attached antimicrobial peptides like neutrophil elastase, which have been shown to trap and kill bacteria, but also contribute to the pathogenesis of inflammatory diseases and cancer^16–18^. Additionally, in cases of prolonged inflammatory stimuli like autoimmune diseases or cancer, neutrophils acquire a distinct phenotype termed granulocytic myeloid-derived suppressor cells (PMN-MDSCs). These cell are considered to promote tumor growth by providing a immunosuppressive tumor microenvironment, yet in cases of autoimmune diseases they have been shown to impair diseases progression^19,20^.

### Metabolic control of neutrophil differentiation

**Figure 1:**
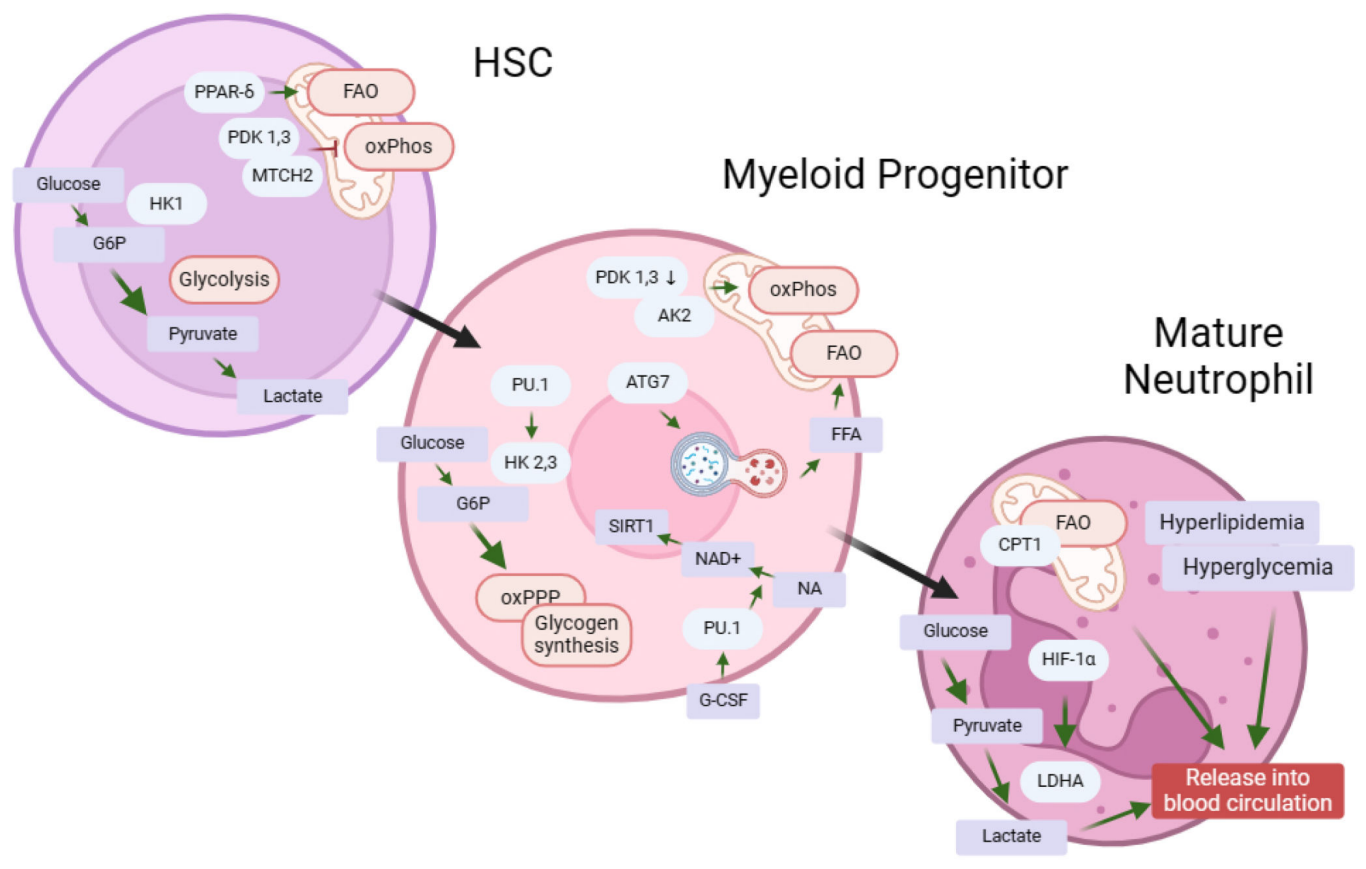
Metabolic control of neutrophilic development in the bone marrow. Due to tissue hypoxia in the HSC compartment, energy is largely produced via glycolysis, while oxPhos is impaired. Additionally, FAO is increased, which is needed for controlled, symmetrical division of HSCs and further myeloid differentiation. In the next step of differentiation, myeloid progenitors increase glycogen synthesis and activity in the oxPPP. Additionally, activity of oxPhos in the progenitor cells is increased. Via the autophagolysosome FFA are generated, which are further utilized for energy production via FAO. G-CSF upregulates NAMPT, which converts NA to NAD+ and subsequently SIRT1. This metabolic process drives neutrophilic maturation. Subsequently, mature neutrophils are released into the bloodstream from the bone marrow due to metabolic adaptations. Both hyperlipidemia and hyperglycemia increase bone marrow release of neutrophils. Additionally, in cases of infections, neutrophils in the bone marrow secrete high levels of lactate, that leads to accelerated release into the peripheral blood. Furthermore, FAO facilitates proper migration from the bone marrow to tissues during infections. Abbreviations: AK: adenylate kinase 2, ATG7: autophagy related 7, CPT1: carnitine palmitoyl transferase 1, FAO: fatty acid oxidation, FFA: free fatty acid, G-CSF: granulocyte colony-stimulation factor, HIF-1α: hypoxia-inducible factor 1α, HK: hexokinase, LDHA: lactate dehydrogenase, MTCH2: mitochondrial carrier homologue 2, NA: nicotinamide, NAD+: nicotinamide adenine dinucleotide, NAMPT: nicotinamide phosphoribosyltransferase, oxPhos: oxidative phosphorylation, oxPPP: oxidative pentose phosphate pathway, PDK: pyruvate dehydrogenase kinase, PPAR-δ: peroxisome proliferation-activated receptor-δ, SIRT1: sirtuin-1

Neutrophils are derived from hematopoietic stem cells (HSC), that differentiate into multipotent progenitor cells (MPP) and subsequently into common myeloid progenitor cells (CMP). As a next step, CMPs differentiate into granulocyte-monocyte progenitors (GMP), that can commit to the neutrophil lineage upon stimulation with granulocyte colony-stimulating factor (G-CSF)^21,22^. On a transcriptional level, this process is regulated by the transcription factors PU.1, C/EBPα, and GFI1^23–25^. Metabolically the transition from HSC to myeloid progenitors is distinctly controlled (Figure 1). Especially in the early steps this is regulated by hexokinase (HK) isoforms. Hexokinase is the first step of glycolysis and catalyzes the phosphorylation of glucose to glucose-6-phosphate. While the isoform HK1 is expressed in HSC and has catabolic properties by providing glucose for glycolysis and ATP generation, CMPs upregulate HK2 and HK3, which have been described as an anabolic enzyme shuttling Glucose-6-Phosphate (G6P) into the pentose phosphate pathway (PPP) or glycogen storage^26,27^. The importance of HK isoforms underpins the central role for glucose metabolism in granulopoiesis, yet it indicates that while HSCs utilize glucose directly for ATP production, myeloid progenitors rely on it for anabolic usage. Additionally, the close connection between glucose metabolism and neutrophil development can also be understood as the transcription factor PU.1 is crucial for neutrophil development by regulating the expression of HK3^28^. In this process, however, not only glucose metabolism is altered upon differentiation but also oxidative phosphorylation (oxPhos). In the case of HSC, it has been described that due to the highly hypoxic environment in the bone marrow, oxPhos is impaired by pyruvate dehydrogenase kinase (Pdk) 1 and 3 dependent mechanisms resulting in an increase in anaerobic glycolysis, which results in accumulation of lactate. However, upon differentiation Pdk1 and 3 expression is downregulated and thereby myeloid progenitors can utilize pyruvate after conversion into acetyl-CoA in the tricarboxylic acid (TCA) cycle and oxPhos^29^. The importance of this balance between glycolysis and oxPhos has also been demonstrated in genetic knock-out models of pyruvate kinase M2 (PKM2) and lactate dehydrogenase A (LDHA) which did not alter HSC to myeloid progenitor differentiation at steady state but were crucial regulators for the efficacy of experimental bone marrow transplantation^30^. In addition to that, mitochondrial carrier homologue 2 (MTCH2), which is a negative regulator of oxPhos, is crucial for HSC homeostasis as a knock-out of it fuels hematopoiesis, an effect that can be mimicked by using G-CSF^31^. Two examples highlighted how systemic glucose abundancy can alter neutrophilic granulocyte differentiation: on the one hand, in two types of murine models for diabetes, which show a high fasting glucose level, GMP numbers in the bone marrow and neutrophils counts in the peripheral blood are clearly increased, which depends on neutrophil derived S100A8/9^32^. On the other hand, by blocking glycolysis with the glucose analog 2-desoxyglucose (2-DG), neutrophils in peripheral blood are significantly decreased in number^33^. Further genetic knock-out models provided insight that glucose uptake via the glucose transporter 1 (GLUT1) is essential for proper development of neutrophils^34^.

Mice harboring a knock-out in the genes encoding for cholesterol transporters ABCA1 and ABCG1 show an increase in GMPs in both blood and the spleen. This depends on G-CSF in the serum upon production by splenic macrophages and dendritic cells via interleukin 23 (IL-23)^35^. Deficiencies in these genes are also shown to decrease neutrophil apoptosis^36^. Another molecular insight on the contribution of lipids and fatty acid oxidation for regular HSC and myeloid progenitor development comes from knock-out models for peroxisome proliferation-activated receptor-δ (PPAR- δ), which tightly regulates HSC maintenance and further development of myeloid progenitors. Both deletion of PPAR-δ and FAO inhibition lead to a symmetric division of HSC causing HSC exhaustion, while FAO activity is crucial for homeostatic, asymmetric division of HSC cells and subsequent neutrophil generation^37^. The usage of fatty acids by neutrophils has also been linked to the balance between glycolysis and oxPhos. Mice deficient in Atg7, a component of the autophagic machinery, which is crucial to break down lipids and supply them for fatty acid oxidation, have a significant increase of neutrophils in the bone marrow. These cells, however, fail to differentiate completely, which can be visualized by a decrease in lobulated nuclei and granules in these neutrophils. To specifically point this effect to GMPs, a *Cebpa* specific knock-out of Atg7 was used, which excludes any HSCs from the knock-out model. Mechanistically this depends on a shift from glycolysis to fatty acid oxidation, which can be rescued by providing fatty acids^38^. The importance of autophagy in regulating neutrophil development is further elucidated by studying Atg5, another component of the autophagic complex, which negatively regulates neutrophil proliferation and granulopoiesis in the bone marrow^39^. Besides autophagy, fatty acids can also be provided via fatty acid synthase (FAS). In an inducible knock-out model of FAS, which is lethal for mice, mature neutrophils cannot be found in the blood circulation. Interestingly, this is not dependent on the development of neutrophilic progenitors. However, the knock-out increases endoplasmic reticulum (ER) stress, which drives neutrophil apoptosis. The increase in ER stress also leads to an increase in peroxisome derived membrane phospholipids that contain ether bonds. By then using an inducible knock-out model for PexRAP, a peroxisomal protein crucial for ether lipid synthesis, neutropenia is mimicked as compared to the FAS-KO mice^40^. Yet, one must note, that in another knock-out-model for ether lipid deficiency no decrease in neutrophil numbers is observed, indicating that other factors could lead to the observed phenotype, *e*.*g*. accumulation of toxic by-products^41^.

More insight on the metabolic control of neutrophil development arises from a hereditary disease (Table 1) called severe congenital neutropenia (CN), which is defined by extremely low absolute neutrophil counts in peripheral blood (< 0.5 x10^9^ per liter; compared to 2.5 – 5 x 10^9^ per liter in healthy patients). Severe congenital neutropenia patients present with recurring and potentially fatal infections and are prone for myelodysplastic syndromes and acute myeloid leukemia. The underlying genetic defects are very heterogeneous, yet, the most frequent defect is due to an autosomal-dominant defect in ELANE, the gene encoding for neutrophil elastase, but also genes involved in G-CSF production have been reported to cause the disease^42,43^. A study using neutrophil precursors from patients with severe congenital neutropenia showed that upon stimulation with G-CSF CN neutrophils upregulate nicotinamide phosphoribosyl transferase (NAMPT). NAMPT is the rate limiting step for the conversion of nicotinamide to nicotinamide adenine dinucleotide (NAD+), which is a central coenzyme for redox reactions. The activation of NAMPT is able to trigger myeloid differentiation by an upregulation of the transcription factor C/EBP-β, a pathway that has been identified in “emergency granulopoiesis”, meaning an accelerated production of neutrophils in cases of infections^44^. By using Vitamin B_3_, which is a precursor for NAD+, neutrophil counts are elevated in healthy patients, making it an interesting therapeutic options for CN patients^45^. Additionally, a mutation in the AK2 gene, which encodes for Adenylate kinase 2, has been reported as a cause for CN^42^. AK2 is a central protein for mitochondrial metabolism and regulates the transfer of the terminal phosphate group between ATP and AMP. Mechanistically, this is studied in patients suffering from reticular dysgenesis (RD), which is caused by an AK2 deficiency^46,47^. AK2 deficient neutrophils have defects in differentiation and survival, and they accumulate lactate and pyruvate. This lactate and pyruvate accumulation is also accompanied by a defect in oxidative phosphorylation, indicating the importance of mitochondrial metabolism for neutrophils differentiation^48^. Similar effects are observed in neutrophils treated with an inhibitor of oxPhos^49^. Additionally, it was shown that the mitochondrial protein HCLS1-associated protein X1 (HAX1) also governs sufficient neutrophil differentiation by controlling proteostasis^50^.

### Fueling the metabolic demands at steady state

#### Glucose

Neutrophils were initially primarily studied as the so called first line of defense against invading microbes. This understanding is also crucial in the description of the metabolic control of neutrophil effector functions. Although neutrophils, like most other mammalian cells, have the capability to utilize carbohydrates, fats, and proteins to fulfill their metabolic demands, glucose has been considered the by far most important fuel for decades. Conceptually this can be explained for two reasons: ATP generation via glycolysis is an extremely fast process, which can therefore meet the quick energy demands by neutrophils in the case of an infection^8^. Additionally, glycolysis can be used for energy production both in cases of normoxia and hypoxia, as is usually found in inflamed tissues^51^. Glycolysis takes place in the cytosol and generates a total of 2 ATP and 2 NADH molecules by converting glucose to 2 pyruvate molecules (Figure 2.1). In steady state, resting neutrophils express the class I glucose transporters GLUT1, 3, and 4, which are upregulated upon activation with TLR agonists and lead to an increase of glucose influx^52,53^. Phagocytotic neutrophils accumulate high levels of lactate, further indicating the importance of glycolysis^54^. A recent report has challenged the long-standing dogma, that neutrophils fulfill their metabolic demands by glucose uptake only. The perception was that upon glucose uptake energy is mainly created via glycolysis and to a small amount stored as glycogen. By using radioactively labeled ^13^C-Glucose tracing experiments, however, it was shown that in cases of acute inflammation neutrophils can not only build glycogen storage via glycolysis but also need sufficient levels of gluconeogenesis via non-glucose substrates like glutamine or palmitate^55^. This process is crucial for killing pathogens and survival. In cases of chronic inflammation, however, gluconeogenesis is impaired, which leads to deficient bacterial killing and a decrease in neutrophil survival^55^. Similar observations were made in neutrophils from guinea pigs upon an inflammatory stimulus in tissue but not peripheral blood^56^. This work demonstrates how neutrophils are able to adapt to settings of low nutrient abundance.

Further evidence on the importance of glycogen storage for neutrophil survival is gathered from studying the hereditary disease glycogen storage disease Ib (GSD-Ib). Patients with this autosomal-recessive disease have a deficient glucose-6-phosphate translocase (G6PT), which shuttles glucose-6-phosphate from the cytosol to the ER and is thereby crucial for gluconeogenesis. These patients present beside metabolic disorders with multiple symptoms related to neutrophil effector functions, including recurring infections and inflammatory bowel disease (IBD). Neutrophils of these patients do not differentiate properly, are dysfunctional as they show impaired chemotaxis and phagocytosis, and have faster rates of apoptosis due to an increase in ER stress^57–59^. On a molecular level, it has been shown that an accumulation of a glucose analog impairs the catalytic activity of hexokinases, which are crucial for gluconeogenesis^60^. This molecular insight led to an intriguing report, in which empagliflozin, an inhibitor of the sodium glucose cotransporter 2 (SGLT2), that facilitates re-uptake of the glucose analog, is used as a potential new target for GSD-Ib. By inhibiting SGLT2, the glucose analog is secreted via the urinary tract and both neutropenia and neutrophil dysfunction were ameliorated in a small patient cohort. Thereby showing how metabolism can be targeted to improved immune cell functions in patients^61^. Furthermore, another glycogenolysis enzyme, namely glucose-6-phosphatase (G6Pase), which hydrolyses glucose-6-phosphate to glucose and phosphate is central for maintaining neutrophil effector functions. Patients with a deficiency in the catalytic subunit C3 (G6PC3) present with severe neutropenia^62,63^. Neutrophils from these patients fail to shuttle glucose between the ER and the cytoplasm. This leads to an accumulation of ER stress and an increase in apoptosis via the anti-apoptotic protein Mcl-1^64,65^. Additionally, it was shown that neutrophils of these patients have a differing glycosylation pattern^66^.

#### Pentose Phosphate Pathway (PPP)

The pentose phosphate pathway (PPP) is described as a glucose oxidizing pathway, which happens parallel to glycolysis. Its main function is to provide ribose 5-phosphate for nucleotide synthesis and nicotinamide adenine dinucleotide phosphate (NAPDH) for maintaining cellular redox balance (Figure 2.2). The latter is particularly important in neutrophils as the respiratory burst by neutrophils relies on NADPH^67^. Upon stimulation neutrophils produce a high amount of reactive oxygen species (ROS), which is called the respiratory burst. This ROS production depends on the NADPH oxidase 2 (NOX2) complex, that consists of the cytoplasmatic proteins p47^phox^, p67^phox^, and p40^phox^, and the membrane proteins gp91^phox^ and p22^phox^. After stimulation, NOX2 is activated, and electrons are transported in a NADPH dependent manner across the membrane to produce hydrogen peroxide and ultimately hypochlorous acid. ROS production can be utilized for killing of extracellular bacteria, but also intracellularly in the phagosome^68,69^. To provide enough NADPH for this process, neutrophils shuttle high amounts of their glucose flux in the oxidative PPP. This is outlined by the ability of a specific blocker of glucose-6-phosphate dehydrogenase (G6PD), which is the first reaction of the oxidative PPP, in blocking the respiratory burst in human and murine neutrophils^70^. The mechanism how this NADPH need can be fulfilled has been elucidated in a recent report: after stimulation with various compounds, neutrophils drastically upregulate genes required for the PPP. During the respiratory burst, most of the glucose is used for the oxidative PPP. To maintain high levels of NADPH, it cycles in the PPP via rubulose-5-phosphatate, fructose-6-phosphate (F6P), glucose-6-phosphate-isomerase (GPI), and glucose-6-phosphate (G6P). Inhibition of this cycling by knock-out models or pharmacological inhibition drastically impair neutrophil effector functions in infectious models^71^. However, excessive NOX2 activity is not always of benefit, as it can also lead to tissue damage^72^. Therefore, it was recently shown, that activation of the 6-phosphofructokinase, liver type (PFKL), which dampens shuttling of glycolytic flux through the PPP can suppress the NOX2 dependent burst and thereby reduce tissue damage by neutrophils^73^. Interestingly, in pregnancy, where neutrophils are reported to be crucial regulators of placental development and inhibit exaggerated T cell response^74^, they show a decrease in the respiratory burst capacity, which is explained by a decreased trafficking of G6PD from the centrosomal to a peripheral location within the cell by microtubules. This nicely elucidates the importance of cellular compartmentalization and proximity between proteins, in this case proximity between PPP enzymes and the NOX2 complex in the plasma membrane^75^.

Once more, the physiological importance of the PPP *in vivo* in patients is best outlined by studying hereditary diseases. The best characterized genetic disease affecting neutrophil function is chronic granulomatous disease (CGD) which is caused by a defect in the genes encoding for components of the NOX2 complex, most prominently in the CYBB gene, which encodes for gp91^phox^. This leads to a defect in the respiratory burst capacity of neutrophilic granulocytes and in extreme cases the respiratory burst is fully absent^76^. Patients suffering from CGD usually present with recurring infections at an early age and need to be treated with prophylactic treatment or if this treatment fails with stem cell transplantation^77^. However, patients have also reported non-infectious manifestations in the lung, gut, or liver, indicating that the oxidative burst is also of relevance besides killing pathogens^78^. Besides CGD there is a metabolic hereditary disease which mimics a large proportion of the symptoms and defects in neutrophil effector functions: G6PD-deficiency. G6PD deficient patients usually present with symptoms of anemia, as the defects leads to increase oxidative stress and thereby death of red blood cells^79^. In addition to that, neutrophils derived from a patient with G6PD deficiency fail to produce NADPH via the PPP and have impaired ability to kill catalase positive bacteria via NOX2 derived ROS^80,81^. Moreover, G6PD deficiency was also shown to impair the ability of neutrophils to form neutrophils extracellular traps (NETs)^82^.

#### Mitochondrial metabolism

Mitochondria are usually considered as the central organelles for cellular metabolism. They are needed to provide bioenergy in the form of ATP, but also to maintain redox homeostasis, generate building blocks for other molecules, and degrade waste from metabolic processes^83^. Functionally, however, it has long been believed that mitochondria are not crucial for ATP generation, but rather in controlling apoptosis of neutrophils^84, 85^. The importance of mitochondria in regulating neutrophil apoptosis was also shown by using neutrophils from patients with a hereditary defect in the succinate dehydrogenase B (SDHB). SDHB has a dual role in mitochondrial metabolism, on the one hand it is needed for the oxidation of succinate to fumarate in the TCA cycle and on the other hand is part of the electron transport chain as complex II. The rare patients who are deficient in SDH present with encephalopathy and cardiomyopathy^86,87^. Neutrophils derived from these patients show impaired apoptosis by an uncoupling of the electron transport chain^88^. Yet, more recently it was demonstrated that mitochondrial respiration is in fact crucial for neutrophil effector functions like chemotaxis, cytokine production, NET production or ROS generation^89–91^. One potential explanation of how mitochondria boost neutrophil effector functions without directly generating energy could be via the glycerol-3-phosphate shuttle (Figure 2.3). This mechanism provides NAD^+^ in the cytosol and protons to complex III in the mitochondria by converting dihydroxyacetone phosphate to glycerol-3-phosphate. NAD^+^ can then subsequently be used to promote glycolysis. Indeed, it was shown that complex III in neutrophils is needed to maintain a membrane potential in neutrophil mitochondria and fueling aerobic glycolysis^92^. Besides these indirect effects, mitochondria were also shown to directly fuel neutrophil effector functions. Mitochondria produce ROS (mitROS) as part of oxidative phosphorylation. These mitROS induce NET production independently from NOX2 and via the membrane channel SK3^93^. Interestingly, the production of these NOX2-independent NETs is not associated with cell death^94^.

ATP, that is mainly produced in the mitochondria, and other related precursors like ADP and AMP do not only play a role in fueling cellular energy demands but are also recognized as signaling molecules. These metabolites are sensed by G protein-coupled receptors of the P2Y family and regulate key effector functions of the immune system^95^. Upon stimulation, neutrophils release ATP by upregulating maxi-anion channels, connexin, and pannexin, which are channels for ATP. This released ATP is crucial to fully activate neutrophils via a paracrine loop and the receptors P2Y2 and the adenosine receptor A3. Functionally this mechanism is essential for activation of neutrophils and regulates effector functions like chemotaxis and defense against bacteria^96,97^. Additionally, ATP was shown to regulate cellular apoptosis of neutrophils^98^.

One fascinating, yet with regards to neutrophils barely studied field is mitochondrial transfer. Multiple cells like macrophages, white adipocytes, or osteoblast are able to transfer their mitochondria to other cells, by which the contribute to diseases like stroke, cancer, or ischemia-reperfusion injury^99^. Upon stimulation platelets can secrete high amounts of mitochondria containing microparticles. Extracellular mitochondria can bind to neutrophils, which leads to an increased release of proinflammatory cytokines^100^. If and how this mitochondria transfer affects cellular metabolism of neutrophils, is currently, however, unclear.

#### Lipid metabolism

Although mature neutrophils depend highly on glycolysis, it is becoming more evident that lipids are needed for proper development and to fulfill crucial effector functions in neutrophils^38^. Three categories of lipids are usually classified: fatty acids (FA), phospholipids, and neutral lipids (triglycerides and cholesteryl esters)^101^. Lipid droplets are the central storage organelle and contain a core of neutral lipids, that are capsulated by a phospholipid bilayer^102^. The main type of energy production from lipid metabolism derives from mitochondrial β-oxidation^101^. Neutrophils incubated with oleic acid, but also derived from *in vivo* patient samples, showed accumulation of lipid droplets within the cells (Figure 2.4)^103^. This accumulation of neutral lipids contrasts with lymphocytes, which usually use fatty acids to generate energy from oxidation. It has been hypothesized that this immediate storage of neutral lipids is needed for proper phagocytosis by neutrophils^104^. A defect in the enzyme adipose triglyceride lipase (ATGL), which is the first step to acquire free fatty acids from triglycerides in lipid droplets, leads to a drastic accumulation of lipid droplets, that is not found in other immune cells. This accumulation is even more pronounced in inflammatory conditions and increases chemotaxis and Ca^2+^ signaling. Functionally, fatty acids are needed to produce inflammatory mediators like leukotriene B4 (LTB4)^105^. Neutrophils express the low-density lipoprotein (LDL) receptor (LDLR) and are able to internalize LDL^106^. Cholesterol is especially needed to maintain membrane integrity of neutrophils, which is important for proper neutrophil adhesion to the endothelium^107,108^. Treatment with statins, a class of drugs blocking the 3-hydroxy-3methylglutaryl coenzyme A (HMG-CoA) reductase that is needed for cholesterol synthesis, impairs neutrophil migration *in vivo*^107,108^. Uptake of LDL can induce chemotaxis and increase Ca^2+^ flow, yet, oxidized LDL (oxLDL) can lead to neutrophil apoptosis^109^. In addition, oxLDL has been shown to induce NET formation by neutrophils^110^. Besides maintaining plasma membrane integrity, lipid derivates are crucial for the synthesis of lipid mediators. The most prominent group of lipid mediators are arachidonic acid (AA) derived and contain among other leukotrienes like LTB_4_, 15-Hydroxyeicosatetraenoic acid (15-HETE), or prostaglandins like prostaglandin E_2_ (PGE_2_)^111^. These lipid mediators can both influence other immune cells like T-cells or NK-cells, yet, they can also intrinsically act on neutrophils and influence adherence and chemotaxis^112,113^. Besides regulating the inflammatory response, the neutrophil derived mediator 12-HETE in essential for maintaining homeostasis of alveolar macrophages in adult mice by decreasing senescence in these cells^114^.

In recent years, more and more research has investigated a part of cholesterol metabolism, which can produce immunomodulatory intermediates, namely oxysterol metabolism. These are oxidized metabolites derived from cholesterol and are considered as precursors for bile acids. Yet, these oxysterols are crucial in maintaining total cholesterol homeostasis, including influx and efflux, by acting via the liver X receptors (LXR), the master regulator of cholesterol synthesis sterol regulatory element-binding protein 2 (SREBP2), and the estrogen receptor^115^. Mice that lack the oxysterol receptors LXR α and β show an increase in neutrophil number and turnover in peripheral blood, which was maintained by a decrease of phagocytosis of circulating neutrophils^116^. Besides these indirect effects, however, LXRα also regulates neutrophil migration and effector functions by suppressing cholesterol efflux via the cholesterol transporters ABCA1 and ABCG1^117,118^.

The importance of lipid metabolism for neutrophil functions is further elucidated in patients suffering from a rare disease called Majeed Syndrome. The patients present with an autoinflammatory bone disorder called chronic recurrent multifocal osteomyelitis (CRMO), congenital dyserythropoietic anemia (CDA), and dermatosis. Genetically, these patients have a defect in a member of the LIPIN family, which converts phosphatidic acid to diacylglycerol (DAG) and there contributes to triacyl glyceride synthesis^119^. It has also been reported that these patients have an increase in neutrophil count in peripheral blood and the dermatosis is neutrophil driven^120^. Mechanistically it is not understood how Majeed syndrome affects neutrophil effector functions^121^.

#### Short chain fatty acids (SCFA)

Dietary fibers are metabolized by microbiota in the gut in an anaerobic manner to short chain fatty acids (SCFA), which influence organismal metabolism and immune cell effector functions. The most prominent SCFAs include acetate, butyrate, and propionate, which can directly alter cellular lipid and glucose metabolism as well as effector functions^122^. In the case of butyrate, the best described producers in the human colon belong to the phylum Firmictues^123,124^. Wildtype mice treated with butyrate show impaired granulopoiesis, seen by a decrease in the number of mature and an increase in immature neutrophils. Butyrate is even able to impair G-CSF triggered granulopoiesis. On a transcriptional level, this is accompanied by changes in genes related to degranulation and maturation. Besides these developmental changes, butyrate also significantly impairs ROS-mediated killing of *Pseudomonas aeruginosa*^125^. Furthermore, butyrate also delays neutrophil apoptosis^126,127^. Additionally, butyrate dampens the production of proinflammatory cytokines, NET formation and the migratory capacity of these cells in a histone deacetylase dependent manner^128^. One has to mention, however, that other reports using another model showed that the SCFAs butyrate, acetate, and propionate can actually increase migratory capacity of neutrophils by upregulating L-selectin and β-integrin and the G protein-coupled receptor 43 (GPR43)^129,130^. These effects of microbial derived butyrate can even be utilized as a potential new therapeutic approach using butyrate producing bacteria^129,130^. In an *in vivo* model for *Clostridium difficile* infection, the butyrate producing *Clostridium butyricum* increased neutrophil migration to the colon and increased clearing of the pathogen, further indicating the therapeutic potential of butyrate producing bacteria^131^. In addition to infections, patients suffering from aortic aneurysm were shown to have reduced butyrate levels due to a lack of the butyrate producing *Roseburia intestinalis* (*R. intestinalis*). This leads to NET-dependent inflammation and vascular remodeling by neutrophils. But substituting mice with either butyrate or *R. intestinalis* aneurysm formation was reduced^132^. The SCFA acetate can also increase the migratory capacity of neutrophils and regulate the secretion of inflammatory cytokines. Mechanistically, this is directly regulated via the free fatty acid receptor 2 (FFAR2)^133,134^.

#### Amino Acid Metabolism

Amino acids can be distinguished into essential and non-essential amino acids and can contribute to cellular metabolism via glycolysis via serine or the TCA cycle among others via glutamine, leucine, and valine. Additionally, they are crucial to sustain redox balance of cells and are the central drivers of protein synthesis^135^. Glutamine has been shown to be utilized by neutrophils (Figure 2.5) and slightly increases tumor necrosis factor α (TNFα) production and ROS production^136–138^. Tracing experiments with radioactively labeled glutamine showed that neutrophils utilize glutamine and convert it into glutamate, a process that is more pronounced upon stimulation with lipopolysaccharide (LPS). Moreover, glutamine is able to fuel gluconeogenesis by providing intermediaries for glycolysis and the TCA cycle^55^. In mammalian cells arginine is mainly metabolized via Arginase 1 (Arg1). Upon stimulation, neutrophils express Arg1 and are able to release it, which impairs T-cell proliferation^139,140^. The essential amino acid tryptophan can be utilized via the enzyme indoleamine 2,3 dioxygenase 1 (IDO1). In cases of infection, neutrophils can upregulate IDO1 in an interferon-γ and CTLA-4 dependent manner, a process needed for the proper killing of fungal pathogens^141^. In addition to that, one mechanism how amino acids alter neutrophil effector functions arose from an investigation of female vs. male neutrophils. Interestingly, female neutrophils upregulate genes needed for pyrimidine and tryptophan metabolism, while male neutrophils use higher levels of arginine, proline, and glutathione metabolism^142^. How these discrepancies regulate female vs. male neutrophil effectors functions needs to be further investigated.

#### Oxygen control of neutrophil metabolism

Whenever inflammation occurs in tissue, there is a high chance of it being accompanied by a lack of oxygen. Therefore, hypoxia and inflammation have been termed as two sides of the same coin^143,144^. According to the classic concept of neutrophils as the first line of defense against invading pathogens in inflamed tissues, it seems obvious that oxygen levels can control neutrophil effector functions and their metabolism. Additionally, as neutrophils need oxygen to maintain the respiratory burst, the importance of oxygen in regulating neutrophilic effector functions becomes obvious^7^. Indeed, the transcription factor hypoxia-inducible factor 1α (HIF1α), which regulates cellular adaption to oxygen supply, has been described as a key regulator of neutrophil effector functions and metabolism^145^. Early work showed that *in vitro* neutrophils have a longer lifespan under hypoxic or even anoxic conditions as compared to a normoxic setting, which is regulated via HIF-1α^146,^ ^147^. Interestingly, patients with a heterozygous germline mutation in the von Hippel Lindau protein (pVHL), which negatively regulates HIF-1α abundancy by facilitating it’s ubiquitination, show a similar phenotype as hypoxic neutrophils with impaired apoptosis and increased antibacterial capacity^148^. *In vitro* tracing experiments of neutrophils in hypoxic conditions showed that they are highly glycolytic and can utilize gluconeogenesis to fulfill their metabolic demands at similar levels as compared to neutrophils under normoxia. Interestingly, hypoxic neutrophils show a higher glutamine utilization than normoxic neutrophils. Additionally, neutrophils from individuals, who were exposed to hypoxia due to high altitude, had higher glycogen storage capacity upon LPS stimulation and lower apoptosis rates^55^. The importance of oxygen in regulating glucose metabolism in neutrophils was further shown by using a myeloid specific knock-out in the gene encoding for the prolyl hydroxylase 2 (PHD2), which like pVHL is a negative regulator od HIF-1α. Neutrophils from these mice have an increase in the glycolytic capacity, glycogen storage, and ATP production. Moreover, the neutrophils were more apoptotic and proinflammatory^149^. Glutamine, which has been identified to fuel neutrophilic metabolism under hypoxic conditions, was reported to be provided from an increased uptake of extracellular proteins. These proteins are then further catabolized in the lysosome via the key metabolic regulator mammalian target of rapamycin (mTOR), which is a master regulator of cellular metabolism in immune cells^150^. Blocking this pathway impairs the proinflammatory phenotype of hypoxic neutrophils^151^. Furthermore, this axis between hypoxia and mTOR was also shown to regulate NET formation^152^. An interesting observation was made by studying how hypoxia regulates mitochondrial metabolism in neutrophils. Hypoxia increases the production of mitochondrial ROS (mitROS) by neutrophils, which can stabilize HIF-1α. This release of mitROS was driven by an increase in flux of the glycerol 3-phophate shuttle. Furthermore, it was suggested that mitROS impair PHD2 activity to increase HIF-1α stability and thereby maintain neutrophil survival under hypoxic conditions^153^. In addition to alterations in effector functions by acute hypoxia, it was also reported that an exposure to hypoxia in the past also alters the capacity of the host to survive infections. In fact, preexposure to hypoxia ameliorated the survival in a murine model of *Staphylococcus aureus* infection by suppressing glucose utilization via inhibition of HIF-1α^154^.

#### pH mediated control of neutrophils

Like hypoxia, it has become ever more obvious that a balanced pH is crucial for maintaining tissue homeostasis by immune cells. In the case of an inflamed tissue, a drop in the pH is often observed and directly influences immune cell functions. Sensing of pH and oxygen levels are in fact connected as they both regulate HIF-1α^155^. Cells can regulate their pH by secreting either lactate or protons via channels like NHE1 or MCT4. In the case of neutrophils, reports show that they produce high levels of lactate via glycolysis *in vitro* and *in vivo*. This contributes to the total amount of lactate produced in the bone marrow *in vivo* after LPS injection. After secretion via MCT4, lactate leads to a drastic release of neutrophils from the bone marrow to the blood stream. Lactate production depends on HIF-1α and ROS/NOX2 and leads to neutrophil mobilization via G protein-coupled receptor 81 (GPR81). This mechanism is especially important in cases of bacterial infections^156^. Another mechanism for how pH can be altered was studied *in vitro*. Neutrophils were shown to have distinct patterns of acidification or alkalinization depending on the specific stimulus used. This is mediated via a Na^+^/H^+^ antiport, which alters ROS production upon stimulation^157^. Interestingly, like hypoxia, acidosis can impair apoptosis of neutrophils. Additionally, it was shown that the migratory capacity of neutrophils drastically changes in acidosis and ROS production is decreased. Furthermore, endocytosis of bacteria is increased, yet, bacterial killing impaired in acidosis^157^. NET production, however, depends on an alkalic pH *in vitro*^158^. Additionally, the bacteria derived SCFA succinate was shown to reduce neutrophilic pH and thereby impair oxygen consumption and ROS production^159^.

In summary, although glucose is arguably the main source for neutrophilic metabolism at steady state, every other canonical metabolic pathway is directly or indirectly involved in maintaining cell survival and effector functions.

### Metabolic adaptions by neutrophils in disease

#### Infection

**Figure 3:**
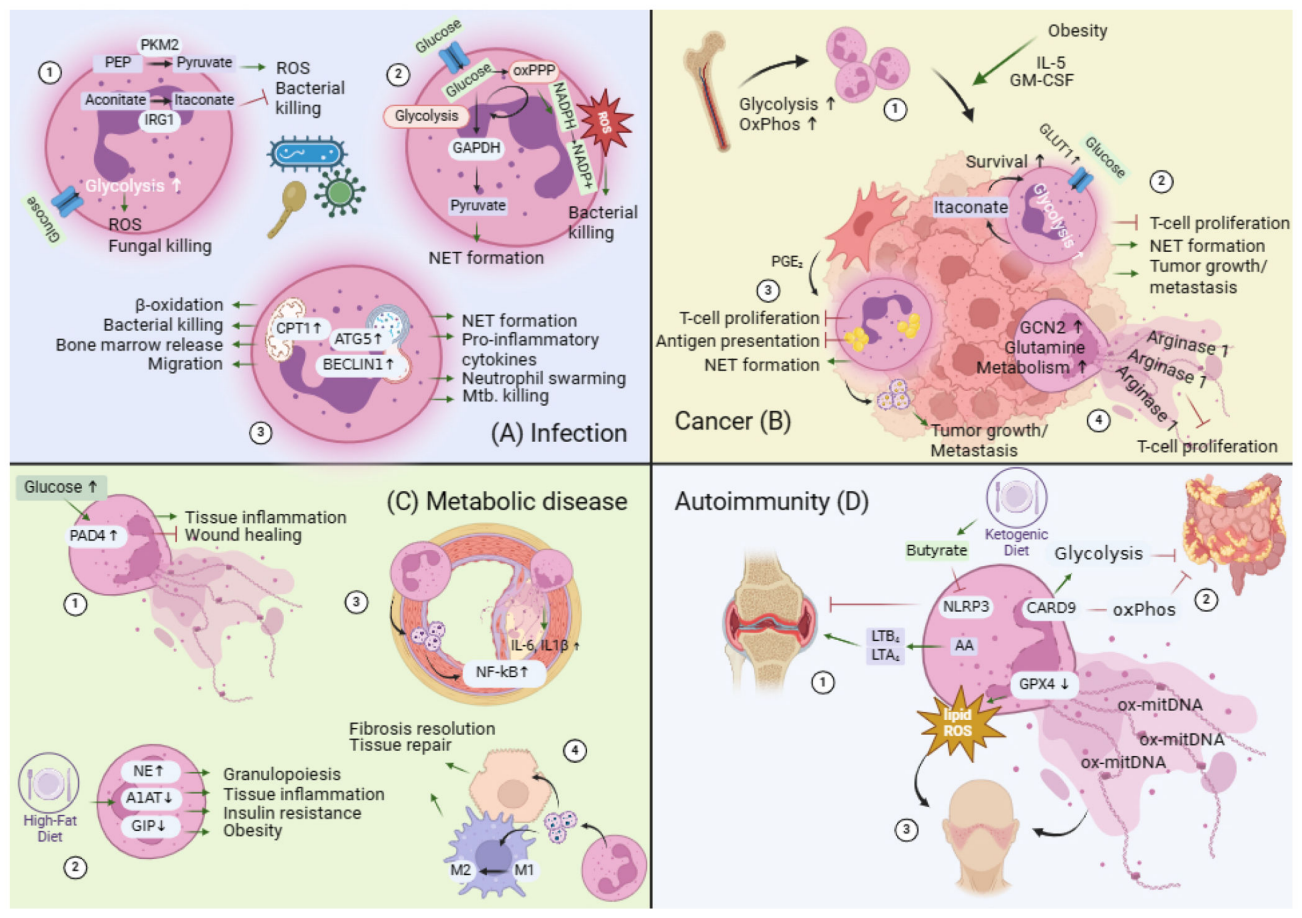
Altered metabolism in the case of diseases. **(A)** (1) Neutrophils, that are phagocytosing a pathogen or are stimulated with a pathogenic compound, increase their glycolytic rate to maintain high levels of ROS for killing of bacteria and fungi. The TCA cycle byproduct itaconate is highly produced by neutrophils in the case of infections and impairs bacterial killing. (2) The PPP provides NADPH for the respiratory burst of neutrophils during infections. To fulfill this need, neutrophils switch on carbon cycling through the oxPPP and decrease glycolysis. (3) Moreover, autophagy and lipid metabolism facilitate the killing of *Mycobacterium tuberculosis*. Release of neutrophils from the bone marrow, migration to the site of infection, and bacterial killing are regulated by β-oxidation. **(B)** (1) Cancer increases release of neutrophils from the blood stream by raising glycolysis and oxPhos. (2) At the tumor site, neutrophils upregulate GLUT1 and increase their glycolytic capacity. Additionally, itaconate is produced in the tumor microenvironment and blocks cell death by neutrophils. (3) Mesenchymal cells promote the accumulation of lipid droplets in neutrophils, which depends on PGE_2_. Lipid droplets can be transferred to tumor cells in extracellular vesicles to support tumor growth. (4) In the tumor microenvironment, neutrophils upregulate GCN2, glutamine metabolism, and Arginase 1, which is localized on NETs. **(C)** (1) In diabetic subjects, a systemic increase in the amount of glucose promotes PAD4-dependent formation of NETs, which impairs wound healing and increases tissue inflammation. (2) Obesity due to a high-fat, western diet alters the transcriptional profile of neutrophils and fuels tissue inflammation and obesity. (3) Neutrophils and NETs are abundant in atherosclerotic lesions. Neutrophil-derived extracellular vesicles are packed with micro-RNA 155, which are taken up by endothelial cells and boosts inflammation via NF-κB. Additionally, NETs in atherosclerotic lesion augment production of the pro-inflammatory cytokines IL-1β and IL-6 by macrophages. (4) Acute liver injury increases the secretion of extracellular vesicles by neutrophils, that contain micro-RNA 223. This process fuels the polarization of macrophages from an M1-like to an M2-like phenotype and increases fibrosis resolution and tissue repair by hepatocytes. **(D)** (1) Inflammatory joint damage in rheumatoid arthritis highly depends on arachidonic acid-derived lipid mediators. A ketogenic diet results in higher levels of the short chain fatty acid butyrate, which blocks the NLRP3 inflammasome and thereby limits joint inflammation due to gout. (2) The metabolic balance between glycolysis and oxPhos is important in limiting inflammatory bowel disease. (3) Patients with systemic lupus erythematosus have neutrophils, that are prone to produce NETs, which contain oxidized mitochondrial DNA. Moreover, lipid ROS accumulates, that promotes neutrophilic cell death and augments disease pathology. Abbreviations: A1AT: alpha 1 antitrypsin, AA: arachidonic acid, ATG5: autophagy related 5, CARD9: caspase recruiting domain 9, CPT1: carnitine palmitoyl transferase 1, GAPDH: glyceraldehyde 3-phosphate dehydrogenase, GCN2: general control nonderepressible 2, GIP: glucose-dependent insulinotropic polypeptide, GLUT1: glucose transporter 1, GM-CSF: granulocyte-macrophage colony-stimulating factor, GPX4: Glutathione peroxidase 4, IL: interleukin, IRG1: immunoresponsive gene 1, LTA2/B4: leukotriene A2/B4, M1: pro-inflammatory macrophage, M2: anti-inflammatory macrophage, NADPH: nicotinamide adenine dinucleotide phosphate, NE: neutrophil elastase, NET: neutrophil extracellular trap, NF-κB: nuclear factor kappa-light-chain-enhancer of activated B cells, NLRP3: NLR family pyrin domain containing 3, oxPhos: oxidative phosphorylation, PAD4: protein-arginine deiminase type-4, PEP: phosphoenolpyruvic acid, PGE2: prostaglandin E2, PKM2: pyruvate kinase isozymes M2, PPP: pentose phosphate pathway, ROS: reactive oxygen species

How neutrophils clear infections has been the most studied area of research in neutrophil biology. More recent work has begun to elucidate how reprogramming of the metabolism during infections determines neutrophil effector functions in this regard (Figure 3.A1)^160^. Phagocytosis or stimulation with the bacterial compound LPS leads to a high glycolytic activity in neutrophils, accompanied by an accumulation of lactate^54,161^. The rate limiting enzyme PKM2 is upregulated in activated murine and human neutrophils and needed for ROS production and antibacterial capacities *in vivo*. PKM2 deficiency leads to a marked decrease in lactate production and – interestingly – a drop in glycerol-3-phosphate levels, which was shown to reduce ROS production, potentially via the glycerol-3-phosphate shuttle^162^. In macrophages, stimulation of LPS leads to a break in the TCA cycle, which causes a drastic increase in the production of itaconate, which has multiple immunomodulatory and antibacterial effects^163^. However, in an *in vivo* mouse model for *S. aureus* infection, neutrophils upregulate the itaconate producing enzyme Irg1 more than any other immune cell. Itaconate impairs glycolysis and ROS production in neutrophils as well as bacterial killing and increases apoptosis^164^. Furthermore, neutrophilic metabolism is not only crucial for the proper elimination of bacteria, but also for the defense against fungi. The fungus *Candida albicans* (*C. albicans*) leads to a higher cell surface expression of the glucose transporter GLUT1 in neutrophils, which increases survival to systemic fungal infections due to boosted fungal killing by neutrophils via ROS^165^. In patients with kidney disease, glucose uptake via GLUT1 is diminished via upregulation of glycogen synthase kinase 3 beta (GSK3β), which leads to systemic *C. albicans* infections and impairs survival^166^. Moreover, glucose metabolism and pathogen defense are linked to mitochondrial metabolism. The protein optic atrophy 1 (OPA1) is one of the key regulators of mitochondrial fusion and fission. Patients with a genetic defect in OPA1 usually present with a variety of neurological symptoms. Interestingly, OPA1 deficient neutrophils also show impaired aerobic glycolysis, which is due to a decrease in the availability of NAD^+^ for glycolysis via impaired complex I activity. *In vivo*, OPA1 deficiency impairs bacterial killing by neutrophils^167^.

As described earlier, the pentose phosphate pathway (PPP) is crucial for mediating neutrophil respiratory burst by providing enough NADPH for the NOX2 complex^71^. By limiting the amount of glucose that can be utilized in the PPP and shuttling it into glycolysis, clearance of *E. coli* is significantly impaired *in vivo*^73^. Similarly, by studying *P. aeruginosa* infection in mice, that were treated with PPP inhibitors, clearance of these bacteria was also diminished^71^. By blocking the PPP via G6PD and 6-phosphogluconate dehydrogenase (6PGD) inhibition, zebrafish infected with the fungus *Aspergillus nidulans* were not able to properly clear the pathogen and had impaired survival^71^.

Interestingly, PPP is not only crucial in the defense against bacteria and fungi but also against viral infections. Neutrophils that were isolated from patients suffering from mild or severe COVID-19 show a drastic increase in metabolites of the PPP. In these patients a decreased activity of GAPDH is also observed, which increases formation of NETs independent of NOX2^168^. Mechanistically, it was not shown how SARS-CoV-2 impairs GAPDH activity, yet, viral glycoproteins can restrict translocation of GAPDH from the cytoplasm to the nucleus, which increases SARS-CoV-2 replication^169^. Alterations in the PPP, however, were not only seen by altering glucose metabolism. Ceramide-1-phosphate (C1P) is a derivative of sphingomyelin that can interact with cytosolic phospholipase A_2_ (cPLA_2_) to produce the lipid mediator prostaglandin E_2_ (PGE_2_). A deficiency of this interaction drastically decreases the oxidative PPP by blocking 6-Phosphogluconate-Dehydrogenase (PGD) and impairs ROS and TNF-α production and NET formation. This lack in the PPP interestingly leads to neutrophilia in a model of *E. coli-*induced peritonitis and ameliorates survival as well as wound healing (Figure 3.A2)^170^.

Carnitine palmitoyl transferase 1 (CPT1), which catalyzes acyl-coenzyme A (acyl-CoA) to acyl-carnitine in the mitochondrial membrane, is the rate limiting enzyme for β-oxidation and linked to control of pathogens. A polymorphism in the CPT1 encoding gene Cpt1a is associated with an increased risk for various infections. In a murine infection model, the CPT1 inhibitor etomoxir impairs the release of neutrophils into the circulation, migration into the lung via paracrine ATP signaling, and ultimately survival. Similarly, patients with the Cpt1a polymorphism present with reduced neutrophils in blood circulation^171^. The protein autophagy related 5 (ATG5) is essential for the formation of the autophagolysosome and thereby for providing lipids. ATG5 knock-out in neutrophils makes mice highly susceptible to *M. tuberculosis* and prevents clearance of the pathogen due to increased release of proinflammatory cytokines and impaired NET formation and neutrophil swarming^172, 173^. Moreover, other factors involved in autophagy like ATG16L1 or BECLIN1 in neutrophils are crucial for proper defense against *M. tuberculosis* as they protect neutrophils from acquiring a transcriptional profile like PMN-MDSC, which was also found in humans infected with *M. tuberculosis*^174^. In addition to cell intrinsic alterations in lipid metabolism, this pathway can also be altered by the uptake of extracellular compounds. Uptake of macrophage-derived exosomes, which contain prostaglandin E_2_ (PGE_2_), leads to a switch in the secretion from pro- to anti-inflammatory lipid mediators and impairs neutrophil ROS production, cell migration of neutrophils, and improves survival against sepsis^175^. Furthermore, neutrophils treated with long-chain fatty acids show increased killing capacity of the malaria-causing pathogen *Plasmodium falciparum (P. falciparum) in vitro*^176^. This might also contribute to the improved survival and decreased bacterial burden in *P. falciparum*-infected mice that are fed a high-fat diet (Figure 3.A3)^177^.

#### Cancer

Neutrophils are being increasingly recognized as crucial players of the immunological tumor microenvironment^178,179^. They are abundantly present in primary tumor sites and were described to acquire a functional phenotype called tumor-associated neutrophils (TANs). TANs can then be further distinguished in a N1 and N2 population. While the N1 population is tumor-cytotoxic and secretes factors like TNF-α, the N2 population is described as a more tumor-promoting population and expresses Arginase^180^. In addition, neutrophils are key drivers of tumor metastasis and might be even more important in this setting than in the primary tumor site^181^. NET formation can activate dormant cancer cells at the site of metastasis and thereby drive the disease^17^. Furthermore, MDSCs, which can have a neutrophilic or a monocytic origin, alter tumor biology by impairing T-cell functions like proliferation and cytotoxicity. Thereby, they support tumor growth and have become an interesting novel target for cancer immunotherapy^20^. Like tumor-associated macrophages^182^, cellular metabolism emerges as a hallmark of neutrophilic effector functions in the tumor microenvironment^178^.

Similar to neutrophils in infections, glucose metabolism drives neutrophil effector functions in the tumor microenvironment (TME). Glycolysis and oxidative phosphorylation fuel migration of neutrophils to the tumor site via autocrine signaling on P2Y and P2X receptors (Figure 3.B1). As soon as they reach the primary tumor site, however, neutrophils and the neutrophilic MDSCs (PMN-MDSC) downregulate glycolysis in the tumor microenvironment^183^. At the tumor site, neutrophils upregulate the glucose transporter GLUT1, which is needed to prolong neutrophil survival in the TME via glycolysis. The upregulation of GLUT1 increases glycolysis in TANs in a murine lung cancer model compared to neutrophils from healthy tissue. This GLUT1 upregulation leads to an accumulation of tumor-promoting Siglec-F^+^-TANs^184^ and reduces the efficacy of radiotherapy^185^. Similarly, in the case of breast cancer-derived liver metastasis, immature neutrophils accumulate in the TME and increase the capacity for glycolysis and oxidative phosphorylation by which they increase NET formation and drive metastasis formation^186^. In addition to the TME, glycolytic neutrophils also influence tumor growth in the periphery. In a mouse model for breast cancer, neutrophils accumulate in the spleen, become highly glycolytic, and increase lactate production. These glycolytic neutrophils in the spleen anergize T-cells by competing for glucose^187^. Glycolytic flux into the TCA cycle in neutrophils results in the accumulation of the TCA cycle derivative itaconate, which can dampen T-cell proliferation by blocking aspartate, serine, and glycine synthesis and by upregulation of PD-L1 and Arginase 1^188^. Itaconate dampens neutrophil ferroptosis due to a GM-CSF, JAK/STAT5, and C/EBPβ signaling cascade and increases the growth of primary and metastatic tumors (Figure 3.B2) ^189^.

Both a high fat diet (HFD) and using genetic obesity models result in neutrophilia in peripheral blood and tissues, thereby showing that lipid abundancy can influence neutrophil function. In obese mice, monocyte-derived GM-CSF and adipocyte-produced IL-5 drive neutrophilia and promote metastasis formation in the lung. Mechanistically, neutrophils drive NET formation in the lung, which boosts metastasis growth^190,191^. Besides hyperlipidemia due to obesity, neutrophils also take up lipids from other sources. In the premetastatic niche neutrophils accumulate high levels of lipids that are stored in lipid droplets by inhibiting the triglyceride hydrolyzing enzyme ATGL in a prostaglandin E_2_-dependent manner. Neutrophils then fuel these lipids to tumor cells to augment their proliferation and metastatic capacity^192^. In the case of tumor relapse after treatment, PMN-MDSCs also transfer lipids to dormant tumor cells to reactivate them. This depends on the acute phase proteins S100A8/A9 that stimulate production of oxidized lipids, which activate tumor cells via production of the fibroblast growth factor receptor 1 (FGFR1)^193^. Additionally, cancer cells can trigger NET formation in a cholesterol-dependent manner via Coiled-Coil Domain Containing Protein – 25 (CCDC25)^194^, which has also been identified as a key-driver of liver metastasis^195^. Moreover, neutrophils in the premetastatic niche produce high levels of the lipid mediators LTB_4_ and LTC/D/E_4_ via the enzyme ALOX5 and thereby drive metastasis growth^196^. In addition, MDSCs numbers in the tumor tissue increase via the adipokine leptin^197^. Interestingly, MDSCs ameliorate metabolic dysfunctions in obese mice and reduce systemic inflammation, yet, in the TME they support tumor growth and block T-cell-mediated tumor cytotoxicity via immune checkpoint blocker PD-L1^198^. One early and crucial regulator of lipid metabolism is the uptake of fatty acids. PMN-MDSCs in the TME upregulate the fatty acid transporter FATP2, which fuels neutrophilic fatty acid oxidation and oxidative phosphorylation, and increases tumor growth by impairing T-cell functions via PGE_2_^199,200^. In addition to fatty acid uptake, the lectin-type oxidized LDL receptor 1 (LOX1, which is also known as oxidized low-density lipoprotein receptor 1), is upregulated by PMN-MDSCs. The increased expression of LOX1 depends on ER stress and was identified as a key driver of PMN-MDSC effector functions^201^. Tumor-derived vesicles can be another source for cholesterol: Tumor cells that lack the transcription factor XBP1, which regulates lipid biosynthesis and is activated as part of the unfolded protein response upon endoplasmic reticulum stress, have a reduction in tumor growth as they secrete less cholesterol containing extracellular vesicles (EVs). These EVs are usually taken up by MDSCs via micropinocytosis and promote their T-cell suppressing functions via STAT3 phosphorylation (Figure 3.B3)^202^. Furthermore, PMN-MDSCs can transfer high levels of oxidized lipids to dendritic cells and block antigen presentation^203^. The cholesterol derivate 22-hydorxycholesterol, a member of the oxysterol family, facilitates recruitment of neutrophils to the tumor, which boosts tumor growth by increasing angiogenesis^204^. Furthermore, pharmacological activation of the liver X receptor (LXR), which is a key target for oxysterols, impairs tumor growth in multiple murine cancer models by inducing apoptosis and impairing the T-cell suppressing functions of PMN-MDSCs. On a mechanistic level this is regulated by apolipoprotein E (ApoE) and low-density lipoprotein receptor-related protein 8 (LPR8) signaling^205^.

Furthermore the oxysterol 27-hydroxycholesterol (27-HC) promotes breast cancer metastasis by attracting neutrophils to the site of metastasis, which is accompanied by a decrease in T-cell numbers^206^.

Amino acid metabolism also affect effector functions of neutrophils in the tumor microenvironment (Figure 3.B4). General control nonderepressible 2 (GCN2) is activated by uncharged transfer RNA (tRNA) in cases of amino acid or glucose starvation. Via its substate eukaryotic translation initiation factor 2 α (eIF2α), GCN2 is a master regulator of metabolism, autophagy, and proliferation. Pharmacological blocking and genetic knock-out of GCN2 drastically reduce tumor growth and boost T-cell activity in multiple murine tumor models, due to impaired PMN-MDSC differentiation and downregulation of PD-L1 and CD206^207,208.^ Moreover, inhibiting glutamine metabolism dampens tumor growth of immunotherapy-resistant breast cancer in mice due to a drastic decrease in the number of PMN- and M-MDSCs in the tumor by limiting G-CSF abundancy, which increases their apoptosis rate^209^. Lastly, NETs from pancreatic ductal adenocarcinoma (PDAC) patients are highly immunosuppressive by expressing different isoforms of Arginase 1^210^.

In summary, glycolysis is needed to maintain neutrophil migration and survival to the tumor site. Additionally, high glycolytic flux functionally impairs T-cell effector functions and thereby boosts tumor growth. Moreover, hyperlipidemia increases granulopoiesis and can both boost tumor cell proliferation and dampen adaptive immunity. IN total this indicates the high translational potential for targeting neutrophilic metabolism in cancer. However, most functional work of neutrophilic metabolism in cancer has been carried out in preclinical murine models. Therefore, more research should strengthen the understanding of metabolic processes in neutrophils in human diseases.

#### Metabolic disease

Diseases that arise from a metabolic pathophysiology like diabetes mellitus (DM) or obesity and its related health consequences are a mounting health issue worldwide^211^. These pathologies have been extensively described to lead to systemic inflammation, which ignites a vicious circle that further exacerbates the diseases^212^. Central drivers of this process are neutrophils.

#### Diabetes

Diabetes comprises pathophysiologically different entities that result in dysregulated blood sugar levels. Type II diabetes (T2D) is the most common type of diabetes and is the result of β-cell dysfunction in the pancreas, insulin resistance, and inflammation^213^. Type I diabetes (T1D), however, is a disease usually found in young patients due to an autoimmune-mediated destruction of the pancreatic β-cells. Like T2D this leads to insulin resistance and dysregulation of the blood glucose levels. Diabetic patients usually suffer from wound healing defects, retinopathy, or cardiovascular diseases and require persistent therapy and monitoring^214^. Neutrophils from patients with T2D are more primed for an enhanced respiratory burst, due to an increased translocation of components of the NOX2 complex to the plasma membrane^215^. In contrast, neutrophils from T1D patients have an impaired respiratory burst, which might indicate a different contribution of neutrophils to these diseases^216^. In non-obese diabetic (NOD) mice, which is a murine model for T1D, neutrophils leave the blood circulation and enter the pancreas at very early stages of development via the macrophage-and β-cell-derived CXCR2 ligands CXCL1 and 2^217^. Furthermore, T2D patients have elevated serum levels of NET components and neutrophils from these patients produce higher levels of NETs upon stimulation *in vitro*^218^. Neutrophils from two different murine T2D models show increased NET formation upon stimulation dependent on PAD4, resembling their behavior in human diabetes patients. Wounds of NOD mice accumulate high levels of NETs that impair their wound healing ability and can be targeted by PAD4 inhibitors to promote healing (Figure 3.C1)^219^. The amino acid intermediate homocysteine is elevated in T2D patients and associated with insulin resistance. *In vitro* homocysteine induces NET formation, correlates with NET components in serum of T2D patients and has a synergistic effect with elevated glucose levels on NET formation ^220^. In ischemic brain injury due to hyperglycemia in mice, NETs are abundantly present and lead to a drastic increase in tissue inflammation. Pharmacological blocking of NET formation ameliorates disease pathology in mice^221^.

#### Obesity

Hypercholesterinemia in mice leads to an increase in neutrophil counts in peripheral blood due to an increased granulopoiesis and epigenetic reprogramming of myeloid precursors via NLRP3 in the bone marrow^222–224^. In accordance, patients undergoing rapid weight loss due to bariatric surgery show a drastic decrease in circulating neutrophils and subsequently tissue inflammation^225^. In addition to the quantitative increase, neutrophils adapt their effector functions in obesity and show increased adhesion to endothelial cells as a result of CXCL1 induced hypercitrullination of histone H3^226^. Neutrophils also increasingly infiltrate the adipose tissue of high-fat diet (HFD) fed mice via a direct contact to adipocytes^227^. Female obese patients undergoing bariatric surgery for weight reduction in fact show decreased chemotaxis of neutrophils as well as reduced NET formation, indicating similar mechanisms operative in murine and human subjects^228,229^. Human overweight and obese patients show increased expression of the bona fide neutrophil activation markers neutrophil elastase (NE) and myeloperoxidase (MPO) in blood leukocytes^230^. NE triggers granulopoiesis in HFD-fed mice via C/EBPα signaling^231^. In obese mice the negative regulator of neutrophil elastase α1-antirypsin (A1AT) is downregulated leading to an increase of NE in the serum. Similarly, in human obese patients A1AT negatively correlates with BMI^232^. A murine knock-out of NE protects from obesity and shows an ameliorated metabolic phenotype including decreased triglyceride levels, smaller fat pad size, a better energy exposure, and ameliorated insulin sensitivity by decreased degradation of the insulin receptor substrate 1 (Irs1). Furthermore, NE knock-out mice show lower numbers of neutrophils and macrophages in the white adipose tissue, indicating lower tissue inflammation. A similar phenotype is observed in mice that overexpress A1AT^232,233^. Besides NE there is also a link between the incretin glucose-dependent insulinotropic peptide (GIP), neutrophils, and obesity. Knock-out of GIP in bone marrow cells triggers increased granulopoiesis and upon HFD also increased influx of neutrophils into the WAT, which highly express S100A8/A9. While HFD fed mice with GIP knock-out bone marrow become obese, this is not seen in mice with a double knock-out in GIP and S100A8/A9. However, in the double knock-out animals, neutrophils counts in blood and WAT are also reduced (Figure 3.C2)^234^.

#### Atherosclerosis

Neutrophils are described as one of the first cell types to infiltrate atherosclerotic plaques, a process that depends on chemokine receptors like CXCR2 and is facilitated by a circadian rhythm^222,235^. Furthermore, hypercholesterinemia induces formation of neutrophil-derived microvesicles (NEV) that can bind to endothelial cells especially at sites of atherosclerosis. NEVs are internalized by endothelial cells and activate them, which further fuels atherosclerosis progression in a NF-κB-dependent manner^236^. Additionally, NETs can be found in human atherosclerosis samples^237^. NET formation is triggered by cholesterol crystals and drives atherosclerosis by priming IL-1β and IL-6 production by macrophages and via an IL-17-dependent positive feed-back loop to further worsen the disease (Figure 3.C3)^238^. One additional driver of NET formation in atherosclerosis are mitochondrial reactive oxygen species, which particularly fuel the disease in aged individuals^239^.

#### Metabolic dysfunction-associated steatotic liver disease (MASLD)

Metabolic dysfunction-associated steatotic liver disease (MASLD), previously known as non-alcoholic fatty liver disease (NAFLD), is an increasingly common metabolic disease, that is the most prevalent type of chronic liver disease. Over the course of the disease MASLD can progress to metabolic dysfunction-associated steatohepatitis (MASH, previously known as NASH), cirrhosis, and ultimately hepatocellular carcinoma (HCC). One of the crucial drivers of these processes is tissue inflammation, exerted among other cells by neutrophils, which are described as an increasingly important target to promote tissue resolution^240^. In murine models, MASLD and MASH are usually modeled by feeding mice a methionine/choline deficient diet (MCD), which leads to a similar liver pathophysiology as in humans. Sufficient migration of neutrophils to the damaged liver is mediated via a signaling axis of TL2/S100A9/CXCL2^241^. In early stages of the disease, pharmacological depletion of neutrophils and of NE limit the disease in MCD-fed mice potentially due to a normalization of the ceramide metabolism in the liver^242,243^. NE is also elevated in the serum of human MASLD patients^244^. In a model of high fat diet-mediated MASLD, neutrophil depletion also ameliorates the disease by promoting mitochondrial biogenesis in the liver and limiting macrophage infiltration^245^. Furthermore, a genetic knock-out of the proinflammatory molecule lipocalin-2 (LCN2), which is highly secreted by neutrophils in MASLD mice and in human patients, ameliorates disease^246^. In MCD-fed mice, neutrophils form NETs in the liver and contribute to tissue inflammation. NETosis is induced via sphingosine-1-phosphate (S1P) and the S1P_2_ receptor, which impairs apoptosis in neutrophils and results in an induction of NETosis^247^. In addition, polyunsaturated fatty acids (PUFA) like linolenic acid that are increased in NASH patients and MCD-fed mice can also trigger NET formation and thereby contribute to disease progression^248^. In a murine HCC model, NETs and regulatory T cells (Treg) positively correlate, of which the latter boost tumor growth by creating an immunosuppressive tumor microenvironment. T cells treated with NETs *in vitro* lead to a shift of CD4 effector T cells to Tregs by promoting oxidative phosphorylation in CD4 T cells^249^. In contrast to the above mentioned reports, in a model of acute liver injury neutrophils are needed for efficient wound healing of the fibrotic liver^250^. On a molecular level, neutrophils promote this process of tissue repair by transferring miRNA-223 to macrophages, which switch from a pro- to an anti-inflammatory wound-repairing phenotype, and to hepatocytes, which downregulates proinflammatory cytokine production and boosts fibrosis resolution (Figure 3.C4)^251,252^.

Taken together, systemic metabolic diseases like diabetes or atherosclerosis were shown to drive a highly proinflammatory phenotype in neutrophils. However, one must state that a lot of the publications on the topic did not specifically investigate neutrophilic cellular metabolism in order to show that systemic metabolic disbalance alters neutrophils’ metabolism, which than further drives the disease progression.

#### Autoimmune/autoinflammatory disease

Autoinflammatory and autoimmune disease include a wide spectrum of conditions affecting a high number of patients. Many studies elucidated the contribution of the cellular metabolism in immune cells to influence these inflammatory diseases. One of the most prominent autoimmune diseases is rheumatoid arthritis, which most commonly affects cartilage tissues. A crucial driver of tissue inflammation in murine models for rheumatoid arthritis are neutrophil-derived lipid mediators. Mice that are deficient in the production of LTB_4_ and LTA_4_ do not develop cartilage damage via the G-protein coupled receptor BLT1^253,254^. An elevation in the metabolite uremic acid promotes joint destruction by forming urea crystals, a disease called gout. Feeding mice a ketogenic diet, which is high in fat and low in carbohydrates, prevents the development of gout by increasing the short-chain fatty acid butyrate, which limits the activation of the neutrophilic inflammasome and cytokine production (Figure 3.D1)^255^. Additionally, mice with increased levels of uremic acid show impaired recruitment of neutrophils to sites of sterile inflammation due to decreased adhesion and extravasation of neutrophils to the vessel wall. Uremic acid uptake depends on the urate transporter SLCA29. In human neutrophils hyperuricemia also leads to impaired phagocytosis^256^.

Inflammatory bowel disease (IBD) is an umbrella term for the diseases Crohn’s Disease (CD) and Ulcerative Colitis (UC). These autoinflammatory diseases heavily rely on metabolic adaptions by immune cells^257^. One key feature of IBD is an acidic pH in the tissue. Neutrophil-derived adenosine contributes to this process, as it regulates the expression of SLC26A3, that secretes chloride ions in exchange for bicarbonate. In IBD this mechanism is dysregulated, which worsens the disease^258^. Mitochondrial metabolism is an important regulator of neutrophilic functions in IBD. Mice with a neutrophil-specific deletion of the gene caspase recruiting domain 9 (CARD9), which is an adaptor protein in pattern recognition receptors and associated with IBD, worsens an experimental IBD mouse model due to decreased neutrophil numbers. Mechanistically, this can be explained as compared to WT neutrophils, which heavily depend on glycolysis, CARD9 knock-neutrophils have increased energy production in the mitochondria, which leads to an accumulation of mitochondrial ROS that increase cellular apoptosis. In line with these findings, patients with a CARD9 single-nucleotide polymorphism also show increased apoptosis rates in neutrophils (Figure 3.D2)^85^. A similar mechanism is seen in mice with a neutrophil-specific deletion of the lymphotoxin receptor beta (LTRβ), which also aggravates experimental IBD. Neutrophils from these mice also show an increased mitochondrial metabolism, yet, glycolysis is also increased in these mice^259^. Moreover, bacterial-derived SCFAs have been shown to affect mucosal immunity in IBD^260^. Neutrophils show impaired migratory capacity and produce less proinflammatory cytokines upon incubation with the SCFA butyrate, which ameliorates colitis in a mouse model i*n vivo* ^128^.

Systemic lupus erythematodes (SLE) is a multi-organ autoimmune disease that especially affects young women and can lead to symptoms like joint inflammation, rash, or pericarditis. This disease is among others heavily driven by neutrophils^261^. Neutrophils from SLE patients show increased oxygen metabolism and ROS production^262^. Additionally, NETs can alter systemic lipid profiles by oxidizing HDL^263^. Moreover, NETs from lupus patients contain mitochondrial protein-oxidized DNA complexes in response to high levels of mitochondrial ROS at steady-state. The oxidized mitochondrial DNA complexes induce the activation of dendritic cells and lead to the production of higher levels of type I interferon that is implicated in disease progression. Application of a scavenger of mitochondrial ROS reduces symptoms in a murine lupus model^90,264^. Accumulation of lipid-ROS and iron overload can lead to cell death by ferroptosis in neutrophils from SLE patients. This process is tightly regulated by the glutathione peroxidase 4 (GPX4) that detoxifies lipid-ROS species. In SLE and murine disease models, reduced GPX4 expression and lipid-ROS accumulation led to the development of neutropenia. Haploinsufficiency of GPX4 alone in mice leads to a lupus-like disease (Figure 3.D3)^264^.

In summary, like metabolic diseases patients suffering from autoimmune or autoinflammatory disorders show a highly proinflammatory phenotype. In the case of IBD it has been shown that mitochondrial metabolism in neutrophils alters disease in preclinical models, while in the case of lupus glutathione homeostasis and ROS drive disease with more indications for relevance in a human setting. However, more research is needed to investigate how these diseases specifically alter cellular metabolism in neutrophils in human disease.

#### Tissue damage

Neutrophil biology in diseases is often considered a double-edged sword as they are crucially needed in the fight against pathogens, yet accumulation of neutrophils can lead to drastic tissue damage and prolong diseases^73^. In polytrauma patients neutrophils upregulate PKM2 indicating high levels of glycolysis^265^. Moreover, in an LPS-induced model of lung injury, PKM2 is also upregulated and a knock-out of this enzyme reduces the disease burden *in vivo*^266^. Neutrophils of stroke patients upregulated PKM2, which leads to hyperactivation of the cells. By deleting PKM2 in myeloid cells, an experimental stroke model is ameliorated due to a reduction in thrombo-inflammation, and the sensorimotor outcome of the mice is improved. PKM2-deficient neutrophils show an increased phosphorylation of STAT3, which has been implicated in MDSC maturation and could thereby also limit tissue inflammation in stroke^267^. Additionally, in a model of heat-induced tissue injury itaconate accumulates in tissue and is mainly derived by mature neutrophils via IRG1. Trauma-derived itaconate gets delivered to the bone marrow, where it alters hematopoiesis and increases the number of granulocyte progenitors^268^. Besides glucose metabolism, cholesterol metabolism is implied in tissue damage by neutrophils. Obese mice show impaired neutrophil migration in LPS-induced lung injury, which leads to amelioration of the disease^269^. In contrast to this observation, the cholesterol lowering drug simvastatin and the PPAR-γ agonist pioglitazone have been shown to also attenuate lung injury by impairing neutrophil migration^270,271^. Additionally, expression of PD-L1, a potent immunosuppressive surface protein, on neutrophils contributes to LPS-induced lung injury by increasing NET formation due to impaired autophagy^272^. Lastly, hypoxia has been shown to regulate how neutrophils mediate tissue damage^273^. *In vitro* studies using the supernatant of hypoxic neutrophils leads to apoptosis of lung epithelial cells and impairs cilia functions^274^. Furthermore, hypoxia extends neutrophils’ lifespan via HIF-1α, a process that is balanced via IL-4 to impair prolonged tissue damage and initiate tissue repair^275^. Moreover, in addition to HIF-1α, HIF-2α is also negatively regulating neutrophil survival in acute lung injury and thereby contributes to disease progression^276^.

## Summary and discussion

This review aimed to summarize the current literature on cellular metabolism in neutrophilic granulocytes for maturation, steady state, and disease. Against old beliefs that neutrophils are only fueled by glycolysis to fulfill their tasks, growing knowledge has emerged implicating various metabolic pathways for neutrophil function. In development neutrophils depend on a balance between glucose and lipid metabolism for proper maturation. Subsequently, they derive large amounts of their energy from glycolysis, yet particularly the pentose phosphate pathway has been shown to be essential for their functionality. Especially in the case of tumor-associated neutrophils, lipid metabolism tightly regulates their contribution to the tumor microenvironment and becomes an increasingly interesting drug target for immunotherapy. So far, only few publications aimed to investigate the intracellular metabolome of neutrophils in a unbiased way^277^, which offers ample future research directions. Although it has been shown that neutrophils can not only be found in the blood but also patrol multiple tissues^14,15^, there is no information how metabolic needs are met by these surveying cells. Moreover, there is a lack of systematic comparisons of tissue specific neutrophil metabolism as it has been done for other cell types^4,278^. With more research focusing on microbiome-targeting therapy for infections^279^ and inflammatory diseases^280^, more research needs to tackle the question how the microbiome affects neutrophils effector functions. The microbiome has been shown to affect aging of neutrophils^281^, therefore, therapies like butyrate producing microbes for infectious or autoinflammatory diseases should also closely be evaluated with regards to their influence neutrophilic effector function. Lastly, especially with regards to tumor-associated neutrophils, more work should analyze on how neutrophils adapt their metabolism in human disease settings to properly evaluate neutrophils as drug target for cancer therapy. Especially primary and metastatic tumors with a high accumulation of neutrophils should investigate a potential cellular crosstalk of neutrophils and tumor cells via metabolites or nutrient adaptations in parallel^282^. Though targeting metabolism in immune cells holds great potential in cancer therapy, one also must keep in mind that pharmacological modulation of neutrophils could just as much reprogram the tumor metabolism or affect other immune cell populations to limit the therapeutic efficacy^283^. Therefore, when aiming to translate metabolic targeting of neutrophils into novel therapies, a holistic understanding of metabolic demands in the TME are needed.

## Methods

The figures were created using BioRender.

## Figures and Tables

**Figure 2:**
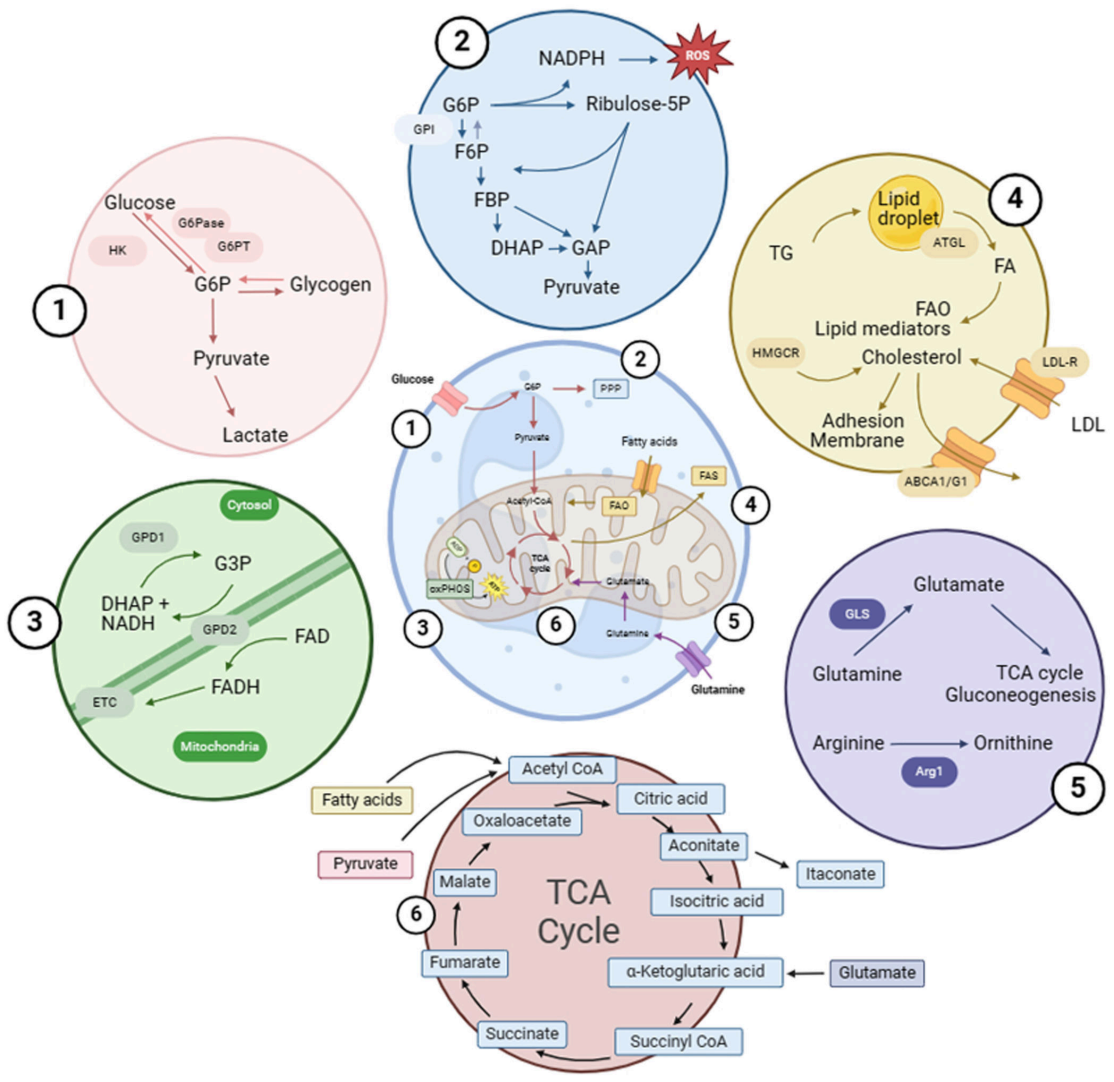
Neutrophil metabolism at steady state. Neutrophilic granulocytes need glycolysis (1), the PPP (2), mitochondrial metabolism (3), lipid metabolism (4), and amino acid metabolism (5) to meet their metabolic demands. **(1)** Glucose can either be used for glycogen synthesis for energy storage or directly for energy production via glycolysis, which leads to an accumulation of pyruvate. If neutrophils undergo phagocytosis, pyruvate is converted to lactate. **(2)** G6P can be shuttled into the oxPPP and further converted into Ribulose-5P, which results in the production of NADPH. The generation of NADPH is especially needed for ROS production via the respiratory burst. Ribulose-5P can either be converted into glycolytic intermediates for energy production or shuttled back into the oxPPP in a cyclic fashion. **(3)** The glycerol-3-phosphate shuttle provides NADH by reducing it to DHAP at the inner mitochondrial membrane. Additionally, this increases energy production via ECT. **(4)** In neutrophils, neutral lipids like triglycerides are mainly stored in lipid droplets, which are a source for FAs. FA can deliver energy via fatty acid oxidation (FAO), are needed for chemotaxis by neutrophils, and are used to produce lipid mediators. Additionally, neutrophils take up LDL via the LDL-R or synthesize cholesterol via the HMGCR. Moreover, cholesterol can be secreted via the cholesterol transporters ABCA1/G1. **(5)** Upon uptake, glutamine is converted to glutamate via the enzyme GLS and used for energy generation via the TCA cycle and for gluconeogenesis. Arginine is converted to ornithine via Arginase 1. **(6)** Energy production via the TCA cycle can be fueled by pyruvate, fatty acids, or glutamate. During inflammation aconitate can be converted to itaconate. Abbreviation: ABCA1/C1: ATP-binding cassette transporter A1/G1, Arg1: arginase 1, ATGL: adipose triglyceride lipase, DHAP: dihydroxyacetone phosphate, ETC: electron transporter chain, F6P: fructose-6-phosphate, FA: fatty acid, FAD: flavin adenine dinucleotide, FAO: fatty acid oxidation, FBP: fructose-1,6-bisphosphate, G3P: glycerol-3-phosphate, G6P: glucose-6-phosphate, G6PD: glucose-6-phosphate dehydrogenase, GAP: glyceraldehyde 3-phosphate, GLS: glutaminase, GPD: G3P dehydrogenase 1, GPI: glucose-6-phosphate isomerase, HK: hexokinase, HMGCR: HMG-CoA reductase, LDL: low density lipoprotein, LDL-R: low density lipoprotein receptor, NAPDH: nicotinamide adenine dinucleotide phosphate, PPP: Pentose phosphate pathway, Ribulose-5P: Ribulose-5-phosphate, ROS: reactive oxygen species, TCA cycle: tricarboxylic acid cycle, TG: triglycerides.

**Table 1:** Hereditary defects interfering with neutrophil metabolism. Further details are outlined in the text.

Pathway	Mutation	Disease	Neutrophil Phenotype	Symptoms
**Glucose**	G6PT	Glycogen storage disease- Ib	Impaired proliferation, impaired phagocytosis	Hypotonia, Infections, IBD
	G6PC3	G6PC3 deficiency	Increased apoptosis	Infections, diarrhea
**PPP**	among others CYBB	Chronic granulomatous diseases	Impaired respiratory burst, impaired killing ability	Infections
	G6PD	G6PD deficiency	Impaired bacterial killing, impaired NETosis	Jaundice, anemia, acute kidney injury
**Lipid metabolism**	LPIN2	Majeed Syndrome	? (inflammatory)	Osteomyelitis, anemia, dermatosis
**Oxygen**	pVHL	von Hippel-Lindau-Syndrome	Impaired apoptosis, increased antibacterial activity	Visceral cysts, benign tumors
**NAD biosynthesis**	among others ELANE	Congenital neutropenia	Impaired differentiation	Infections
**Mitochondria**	AK2	Reticular dysgenesis	Impaired differentiation	Sensorineural deafness, Infections
	SDHB	SDHB deficiency	Impaired apoptosis	Paraganglioma, encephalopathy, cardiomyopathy

## Summary Sentence

We discuss how neutrophilic metabolism adapts during development, which metabolic pathways fuel their functionality and how these processes are reconfigured in case of various diseases.

## Abbreviation List

15-HETE: 15-Hydroxyeicosatetraenoic acid
27-HC: 27-hydorxycholesterol
2-DG: 2-desoxyglucose
6-phosphogluconate dehydrogenase: 6PGD
A1AT: α1-antirypsin
AA: arachidonic acid
ABCA1/G1: ATP-binding cassette transporter A1/G1
acyl-CoA: acyl-coenzyme A
ADP: Adenosine diphosphate
AK2: Adenylate kinase 2
ALOX5: arachidonate 5-lipoxygenase
AMP: Adenosine monophosphate
ApoE: apolipoprotein E
Arg1: arginase 1
ATG: autophagy related
ATGL: adipose triglyceride lipase
ATP: adenosine triphosphate
C/EBPα: CCAAT-enhancer-binding protein alpha
C1P: seramide-1-phosphate
CARD9: caspase recruiting domain 9
CCDC25: Coiled-Coil Domain Containing Protein – 25
CD: Crohn’s disease
CDA: congenital dyserythropoietic anemia
CGD: chronic granulomatous disease
CMP: common myeloid progenitor
CN: congenital neutropenia
COVID-19: coronavirus disease 2019
cPLA_2_: cytosolic phospholipase A_2_
CPT1: Carnitine palmitoyl transferase 1
CRMO: chronic recurrent multifocal osteomyelitis
CTLA4: cytotoxic T-lymphocyte associated protein 4
CXCL: C-X-C motif ligand
CXCR: CXC chemokine receptors
CYBB: cytochrome b (558) subunit beta
DAG: diacylglycerol
DM: diabetes mellitus
ER: endoplasmic reticulum
EV: extracellular vesicle
F6P: fructose-6-phosphate
FA: fatty acid
FAS: fatty acid synthesis
FFAR2: free fatty acid receptor 2
G6P: glucose-6-phosphate
G6Pase: glucose-6-phosphatase
G6PC3: glucose-6-phosphatase catalytic subunit 3
G6PD: glucose-6-phosphate dehydrogenase
G6PT: glucose-6-phosphate translocase
GAPDH: glyceraldehyde 3-phosphate dehydrogenase
GCN2: general control nonderepressible 2
G-CSF: granulocyte colony-stimulating factor
GFI1: growth factor independent 1 transcriptional repressor
GIP: glucose-dependent insulinotropic peptide
GLUT1: glucose transporter 1
GMP: granulocyte-monocyte progenitors
GPI: glucose-6-phosphate-isomerase
GPR: G protein-coupled receptor
GPX4: glutathione peroxidase 4
GSD: glycogen storage disease
GSK3β: glycogen synthase kinase 3 beta
HAX1: HCLS1-associated protein X1
HCC: hepatocellular carcinoma
HFD: high fat diet
HIF-1α: hypoxia-inducible factor 1 alpha
HK: hexokinase
HMG-CoA: 3-hydroxy-3methylglutaryl coenzyme A
HSC: hematopoietic stem cell
IBD: inflammatory bowel disease
IDO1: indoleamine 2,3 dioxygenase 1
Irs1: insulin receptor substrate 1
LAD: leukocyte adhesion deficiency
LCN2: lipocalin-2
LDHA: lactate dehydrogenase A
LDL: low density lipoprotein
LDLR: low density lipoprotein receptor
LOX1: lectin-type oxidized LDL receptor 1
LPS: lipopolysaccharide
LTB4: leukotriene B4
LTRβ: lymphotoxin receptor beta
LXR: liver X receptor
MASH: metabolic dysfunction-associated steatohepatitis
MASLD: metabolic dysfunction-associated steatotic liver disease
MCD: methionine/choline deficient diet
Mcl-1: induced myeloid leukemia cell differentiation protein
MCT4: monocarboxylate transporter 4
MDSC: myeloid-derived suppressor cell
mitROS: mitochondrial ROS
MTCH2: mitochondrial carrier homologue 2
mTOR: mammalian target of rapamycin
NAD: nicotinamide adenine dinucleotide
NAFLD: non-alcoholic fatty liver disease
NAMPT: nicotinamide phosphoribosyl transferase
NAPDH: nicotinamide adenine dinucleotide phosphate
NE: neutrophil elastase
NEV: neutrophil -derived microvesicle
NHE1: sodium-hydrogen exchanger 1
NLRP3: NLR family pyrin domain containing 3
NOD mouse: non-obese diabetic mouse
NOX2: NADPH oxidase 2
OPA1: optic atrophy 1
oxLDL: oxidized LDL
oxPhos: oxidative phosphorylation
PAD4: protein-arginine deiminase type-4
PDAC: pancreatic ductal adenocarcinoma
Pdk: pyruvate dehydrogenase kinase
PD-L1: programmed death-ligand 1
PFKL: 6-phosphofructokinase, liver type
PGD: 6-Phosphogluconat-Dehydrogenase
PGE_2_: prostaglandin E_2_
PHD2: prolyl hydroxylase 2
PKM2: pyruvate kinase isozyme M2
PMN-MDSC: granulocytic myeloid-derived suppressor cells
PPAR-δ: proliferation-activated receptor-δ
PPP: pentose phosphate pathway
PUFA: polyunsaturated fatty acids
pVHL: von Hippel Lindau protein
RD: reticular dysgenesis
ROS: reactive oxygen species
S1P: sphingosine-1-phosphate
SARS-CoV-2: severe acute respiratory syndrome coronavirus 2
SCFA: short chain fatty acid
SDHB: succinate dehydrogenase B
SGLT2: sodium glucose cotransporter 2
SK3: small conductance calcium-activated potassium channel 3
SLC: solute carrier family
SLE: systemic lupus erythematodes
SREBP2: sterol regulatory element-binding protein 2
STAT: signal transducer and activator of transcription
T1D: type 1 diabetes mellitus
T2D: type 2 diabetes mellitus
TAN: tumor-associated neutrophils
TCA cycle: tricarboxylic acid
TLR: Toll-like receptor
TME: tumor microenvironment
Treg: regulatory T-cell
tRNA: transfer RNA
UC: ulcerative colitis
